# Neutrophil-to-Lymphocyte Ratio as a Prognostic Biomarker for Patients With Metastatic Renal Cell Carcinoma Treated With Immune Checkpoint Inhibitors: A Systematic Review and Meta-Analysis

**DOI:** 10.3389/fonc.2021.746976

**Published:** 2021-11-11

**Authors:** Xiuqiong Chen, Fanqiao Meng, Richeng Jiang

**Affiliations:** ^1^ Tianjin Medical University Cancer Institute and Hospital, National Clinical Research Center for Cancer, Tianjin, China; ^2^ Key Laboratory of Cancer Prevention and Therapy, Tianjin, China; ^3^ Tianjin’s Clinical Research Center for Cancer, Tianjin, China; ^4^ Department of Thoracic Oncology, Tianjin Lung Cancer Center, Tianjin Cancer Institute and Hospital, Tianjin Medical University, Tianjin, China; ^5^ Department of Hematology, Tianjin Medical University General Hospital, Tianjin, China

**Keywords:** neutrophil-to-lymphocyte ratio, renal cell cancer, immune checkpoint inhibitor, prognosis, biomarker

## Abstract

There is increasing evidence to suggest that the neutrophil-to-lymphocyte ratio (NLR) is related to the prognosis of patients with renal cell carcinoma (RCC) treated with immune checkpoint inhibitors (ICIs). However, these findings are inconsistent. The present study was performed with the aim of exploring the utility of NLR in patients with RCC treated with ICIs. For this purpose, a comprehensive search of PubMed, Web of Science, and Embase was performed to find studies evaluating the prognostic value of NLR. The overall survival (OS) and progression-free survival (PFS) were the assessed clinical outcomes. All statistical analysis was performed using Stata version 12.0 software. The combined hazard ratios (HRs) and 95% confidence intervals (CIs) of NLR for OS and PFS were calculated using the random-effect models. Heterogeneity was evaluated based on the *I* ^2^ value and Cochran’s *Q* test. Egger’s and Begg’s tests were applied to precisely assess the publication bias. The “trim and fill” method was adopted to perform the sensitivity analysis to determine whether the results were stable. In total, 12 studies encompassing 1,275 patients were included in the final analysis. The results revealed that a high NLR at baseline or pre-therapy was associated with a poor OS (HR, 2.23; 95% CI, 1.84–2.70; *p* < 0.001) and PFS (HR, 1.78; 95% CI, 1.72–2.09; *p* < 0.001). During the course of treatment, a decrease in the NLR was associated with a significantly longer OS (HR, 0.34; 95% CI, 0.20–0.56; *p* < 0.001) and PFS (HR, 0.44; 95% CI, 0.30–0.63; *p* < 0.001) compared to an increase in NLR. As a preliminary screening of other risk factors, age, sex, race, and IMDC risk may have a certain prognostic value for RCC treated with ICIs. People over 70 years old had better OS compared to people younger than 70 (HR, 0.65; 95% CI, 0.48–0.89). Non-Caucasians treated with immunotherapy had a worse OS (HR, 8.67; 95% CI, 2.87–26.2) and PFS (HR, 2.65; 95% CI, 1.28–5.48) than Caucasians. Males had a worse OS than females (HR, 1.48; 95% CI, 1.14–1.93). Compared with the IMDC favorable risk group, the OS of the IMDC poor risk group was worse (HR, 2.59; 95% CI, 1.56–4.32). There was no significant publication bias or heterogeneity observed in the present study. On the whole, the present study demonstrated that an elevated NLR is associated with an adverse OS and PFS in patients with RCC treated with ICIs. The NLR may thus be used as a readily available prognostic biomarker for these patients. Age, sex, race, and IMDC risk may have potential predictive value for the prognosis of RCC treated with ICIs. However, further investigations are warranted to validate these results.

## Introduction

According to the 2018 GLOBLE data, 403,000 individuals are diagnosed with kidney cancer each year, accounting for 2.2% of all cancers worldwide (1). The most common subtype of renal cell carcinoma (RCC) is clear cell carcinoma, which accounts for ~75% of all cases (2). RCC accounts for 5% and 3% of all malignancies among adult males and females, respectively. It is the sixth most common type of cancer among males and the ninth among females (3). Approximately one-third of patients with RCC have experienced metastasis by the time of diagnosis (4).

For patients with advanced RCC, the selection of effective treatment options is critical. Recently, several immune checkpoint inhibitors (ICIs) have been shown to be effective against metastatic RCC (mRCC). RCC tissues are infiltrated by a large number of inflammatory cells, such as T cells, natural killer cells, dendritic cells, and macrophages, rendering immunotherapy a possible effective treatment. The Checkmate-025 study revealed that when Nivolumab monotherapy was used in the second- or third-line therapy of mRCC, both programmed death-ligand (PD-L)1(+) and PD-L1(−) patients benefited from immunotherapy (5). However, for first-line therapy, whether PD-L1 expression is positive or negative, patients with mRCC can benefit from treatment with PD-1 monoclonal antibody, such as Pembrolizumab or PD-L1 monoclonal antibody, such as Atezolizumab and Avelumab combined with vascular targeted therapy (6–8). However, in the CheckMate 214 study, 776 subjects were tested for PD-L1 expression. According to PD-L1 expression, stratified analysis found that for patients with PD-L1 ≥1%, the objective response rate (ORR) was significantly higher in the combined treatment group than in the Sunitinib control group (58% vs. 22%; *p* < 0.001), and median progression-free survival (PFS) was extended by 16.9 months [22.8 vs. 5.9 months; hazard ratio (HR), 0.46; 95% confidence interval (CI), 0.31–0.67]. Of note, in terms of patients with PD-L1 <1%, the ORR was still significantly higher in the immune combination group than in the Sunitinib control group (37% vs. 28%; *p* = 0.03), and the difference in PFS was not statistically significant (11.0 vs. 10.4 months; HR, 1.00, 95% CI, 0.80–1.26) (9). Therefore, PD-L1 is not a perfect predictor of clinical outcomes in immunotherapy for RCC. Thus, the identification of factors associated with the efficacy of immunotherapy for mRCC is essential for guiding precise therapy and surveillance of disease.

In recent years, it has become clear that tumor-related inflammatory responses, such as local and systemic inflammation, and decreased or increased myelopoiesis, substantially contribute to the development and progression of malignancies (10). The alteration of peripheral blood biomarkers, such as the neutrophil-to-lymphocyte ratio (NLR) can represent the systemic inflammatory response in patients. Several studies have demonstrated that the NLR is a potent prognostic biomarker related to a worse overall survival (OS) in several tumor types, including mRCC in the pre-immunotherapy era (11–15). Currently, a growing number of peripheral blood biomarkers, particularly NLR, have been found to be associated with ICI treatment outcomes for various types of cancer (16). Inflammatory indicators related to therapeutic efficacy may guide clinical decision-making.

Currently, although several studies have explored the prognostic value of NLR in patients receiving immunotherapy for RCC (17–28), it is still difficult to verify the prognostic role of NLR in patients with RCC treated with ICIs. Certain studies have suggested that the NLR is not associated with the prognosis of patients with RCC treated with immunotherapy (21, 26). Additionally, some of the published studies had a small sample size (17, 18, 28). Hence, the present comprehensive meta-analysis was conducted in order to precisely evaluate the prognostic significance of the NLR in patients with RCC receiving immunotherapy.

## Data and Methods

### Literature *S*earch

A comprehensive search strategy was applied to identify all relevant literature in the PubMed, Web of Science, and Embase databases up to July 2021. The search terms were as follows: “Neutrophil to lymphocyte” OR “inflammatory biomarkers” OR “Immunoinflammatory measures” OR “Inflammatory indices” OR “NLR” OR “Neutrophil-to-Lymphocyte Ratio” AND “PD-L1” OR “PD-1” OR “nivolumab” OR “immune checkpoint blockade” OR “Immune Checkpoint Inhibitors” OR “immunotherapy” AND “renal cancer” OR “kidney carcinoma” OR “kidney cancer” OR “RCC” OR “renal cell carcinoma”. The reference lists of the identified studies were also examined.

### Inclusion Criteria

Studies that fulfilled the following criteria were included: (i) All patients were diagnosed with mRCC according to the current clinical guidelines and treated with ICIs; (ii) the NLR of patients was calculated, and the association between NLR and prognosis was also investigated; (iii) HR values and 95% CIs could be extracted from the studies or described in the studies; (iv) survival information included the OS and PFS; (v) articles were written in the English language.

### Quality Evaluation

The quality assessment methods from Hayden et al. (29) were used in the present study. It was recommended that the quality appraisal of prognostic studies consider six potential biases: Study attrition, study participation, outcome measurement, prognostic factor measurement, analysis and confounding measurement, and account. The evaluation of risk for bias should be completed by at least two independent reviewers. The score for each item in the quality assessment is 0–2, the maximum score for each study is 12 points, and a score of ≥8 is considered high quality.

### Data Extraction

The information obtained from the studies included the year of publication, first author, country, the number of patients, age, sex, histological type, race, Eastern Cooperative Oncology Group (ECOG) status, prior nephrectomy, the number of prior anti-VEGF therapies, International Metastatic RCC Database Consortium (IMDC) risk group, number of metastatic sites, study type, testing time, cutoff value for NLR, and survival outcomes. Survival data included the HR and 95% CI values for OS and PFS. If the HR and 95% CI values could not be directly extracted from the original study, the reported methods from Tierney et al. (30) and Parmar et al. (31) were used to calculate these statistical variables.

### Statistical Analysis

Authoritative statistical software (Stata 12.0: Stata Corporation) was used to perform the meta-analysis. The HR and 95% CI values were applied to estimate the prognostic value of NLR for patients treated with ICIs. Individual HR and 95% CI values were combined to an overall HR and 95% CI. An HR >1 indicated a worse survival for the experimental group, a 95% CI containing the no. 1 and *p* < 0.05 indicated a significant difference statistically between the two groups. The *I* ^2^ statistic and Cochran *Q* test were applied to detect the heterogeneity between studies; *p* ≤ 0.1 and *I* ^2^ > 50% indicated a substantial heterogeneity between studies and random effects models were adopted. When significant heterogeneity existed, subgroup analysis could be employed to identify the source of heterogeneity. Begg’s test, Egger’s test, and visual inspection of a funnel plot were carried out to evaluate the possibility of publication bias. Egger’s test result was the primary indicator, and a symmetry funnel plot with a *p*-value ≥0.05 was considered as an insignificant publication bias.

## Results

### Literature Characteristics

**Figure 1:**
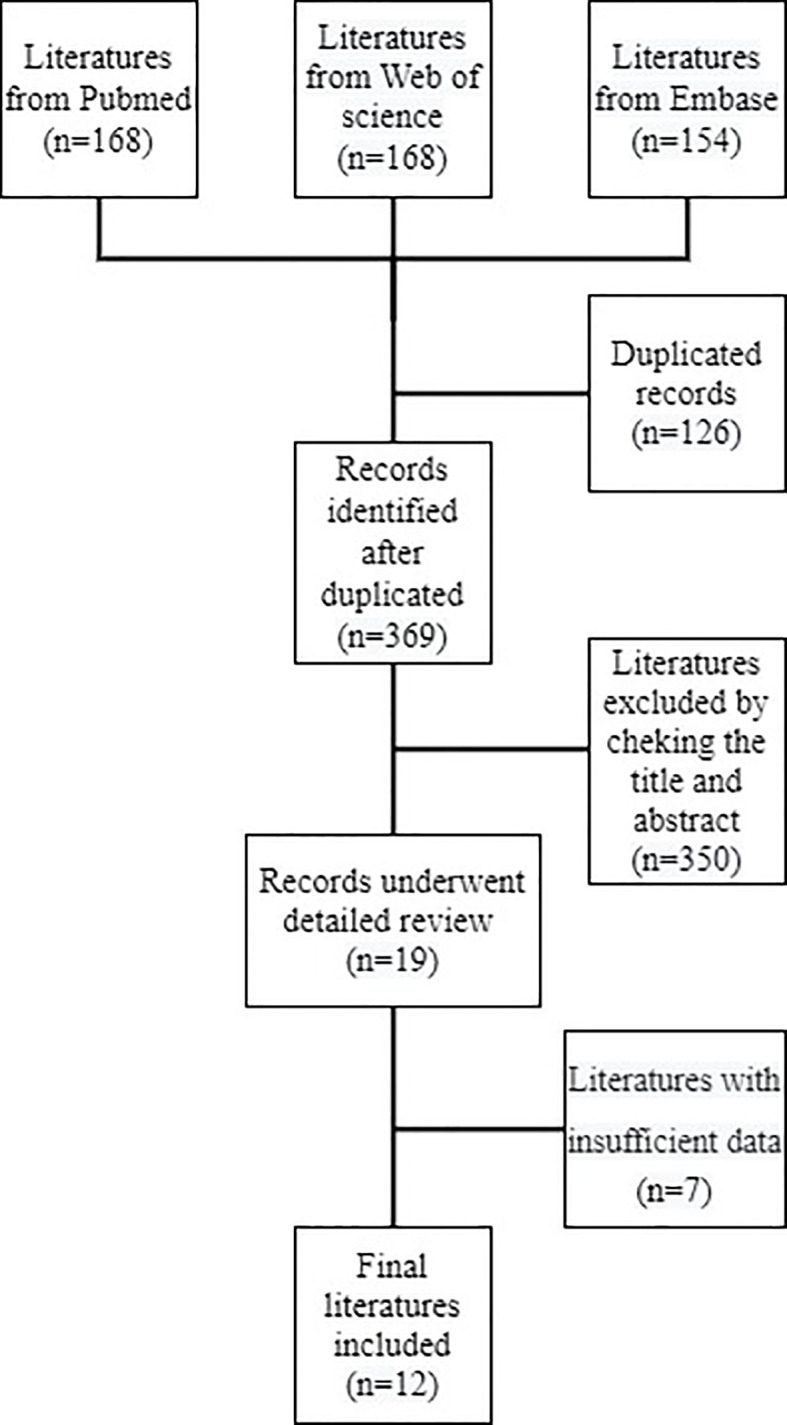
Flow diagram of record selection.

A total of 495 references were collected from PubMed, Web of Science, and Embase. In total, 369 records were left after deleting the duplicates. After examining titles and abstracts, 19 studies were identified. A total of seven studies had insufficient data following a full-text review, leaving 12 records included according to the eligibility criteria (**Figure 1**) (17–28). The literature search was performed by two investigators, and any disagreements between them were settled by consensus. The basic features of the included trials are summarized in **Tables 1**, **2**. The total number of patients from the included studies was 1,275, ranging from 37 to 404 cases per trial. In total, 9 studies were from Western countries, including the USA (17–21, 28), Italy (24, 27), and France (25). Another three were from Japan (22, 23, 26). Only one study was prospective (24) and the remaining studies were retrospective studies. The majority of studies measured the number of neutrophils and lymphocytes at baseline or pre-treatment, and then calculated the NLR; four trials also tested the number under therapy (19, 22, 25, 28). The cutoff values of the majority of studies were 3 (18, 23–28); those in three studies were 3.9 (19), 4.2 (20), and 5.5 (17), and the values in other studies were 5 (21, 22). Both the OS and PFS were evaluated in all nine studies (17–19, 22, 23, 25–28), In total, two studies (21, 24) only had OS and one study (20) only had PFS data available. The scores of quality evaluation for the included trials ranged from 7 to 11; 10 scored >8 (17–20, 22–27), and 2 scored <8 (21, 28).

**Table 1:** Study features of the 12 eligible records.

Author (Year)	Country	Study type	Patients	Testing time	Group	Clinical outcome	Quality score
Asim M (2017)	USA	Retrospective	38	Pretherapy	≥5.5 *vs.* <5.5	PFS, OS	10
Jeyakumar G (2017)	USA	Retrospective	42	Pretherapy	≥3 *vs.* <3	PFS, OS	11
Lalani A (2018)	USA	Retrospective	142	Under therapy	deNLR *vs.* inNLR	PFS, OS	10
				Baseline	≥3.9 *vs.* <3.9	PFS, OS	
Zahoor H (2018)	USA	Retrospective	90	Baseline	≥4.2 *vs.* <4.2	PFS	8
Rohit K (2018)	USA	Retrospective	65	Pretherapy	≥5 vs<5	OS	7
Suzuki K (2019)	Japan	Retrospective	65	Under therapy	deNLR *vs.* inNLR	PFS, OS	9
				Pretherapy	≥5 vs<5	PFS, OS	
Ishihara H (2019)	Japan	Retrospective	58	Pretherapy	≥3 *vs.* <3	PFS, OS	8
Giorgi U (2019)	Italy	Prospective	196	Baseline	≥3 *vs.* <3	OS	9
Simonaggio A (2020)	France	Retrospective	86	Under therapy	deNLR *vs.* inNLR	PFS, OS	10
				Baseline	≥3 *vs.* <3	PFS, OS	
Nishiyama N (2020)	Japan	Retrospective	52	Baseline	≥3 *vs.* <3	PFS, OS	8
Rebuzzi S (2020)	Italy	Retrospective	404	Baseline	≥3 *vs.* <3	PFS, OS	8
Arnab B (2020)	USA	Retrospective	37	Under therapy	deNLR *vs.* inNLR	PFS, OS	7

deNLR, decrease of NLR; inNLR, increase of NLR; OS, overall survival; PFS, progression-free survival.

**Table 2:** The association between other risk factors and overall survival and progression-free survival of patients with renal cell carcinoma treated with immunotherapy.

Factor	Studies number (OS/PFS)	OS		PFS	
		HR	95% CI	HR	95% CI
Gender (male vs female)	5/4	1.48	1.14-1.93	1.10	0.85-1.44
Histologic type (clear cell vs non-clear cell)	4/3	0.84	0.52-1.35	0.82	0.52-1.28
Age (≥70 vs <70 )	4/3	0.65	0.48-0.89	0.73	0.51-1.06
Race (non-caucasian vs caucasian)	1/2	8.67	2.87-26.2	2.65	1.28-5.48
ECOG (0-1 vs 2-4)	1/1	0.42	0.10–1.74	0.46	0.16–1.31
Prior Nephrectomy (yes vs no)	3/3	0.65	0.33-1.29	1.24	0.72-2.12
Number of Prior anti-VEGF Therapies (>1 vs ≤1 )	3/3	1.70	0.98-2.96	1.09	0.75-1.59
IMDC Risk Group (poor vs favorable)	3/4	2.59	1.56-4.32	1.20	0.74-1.94
Number of metastatic sites (≥ 2 vs <2)	3/5	1.11	0.67-1.85	0.98	0.52-1.83

ECOG, Eastern Cooperative Oncology Group; VEGF, vascular endothelial growth factor; IMDC, International Metastatic Renal Cell Carcinoma Database Consortium.

### NLR and OS for RCC

**Figure 2:**
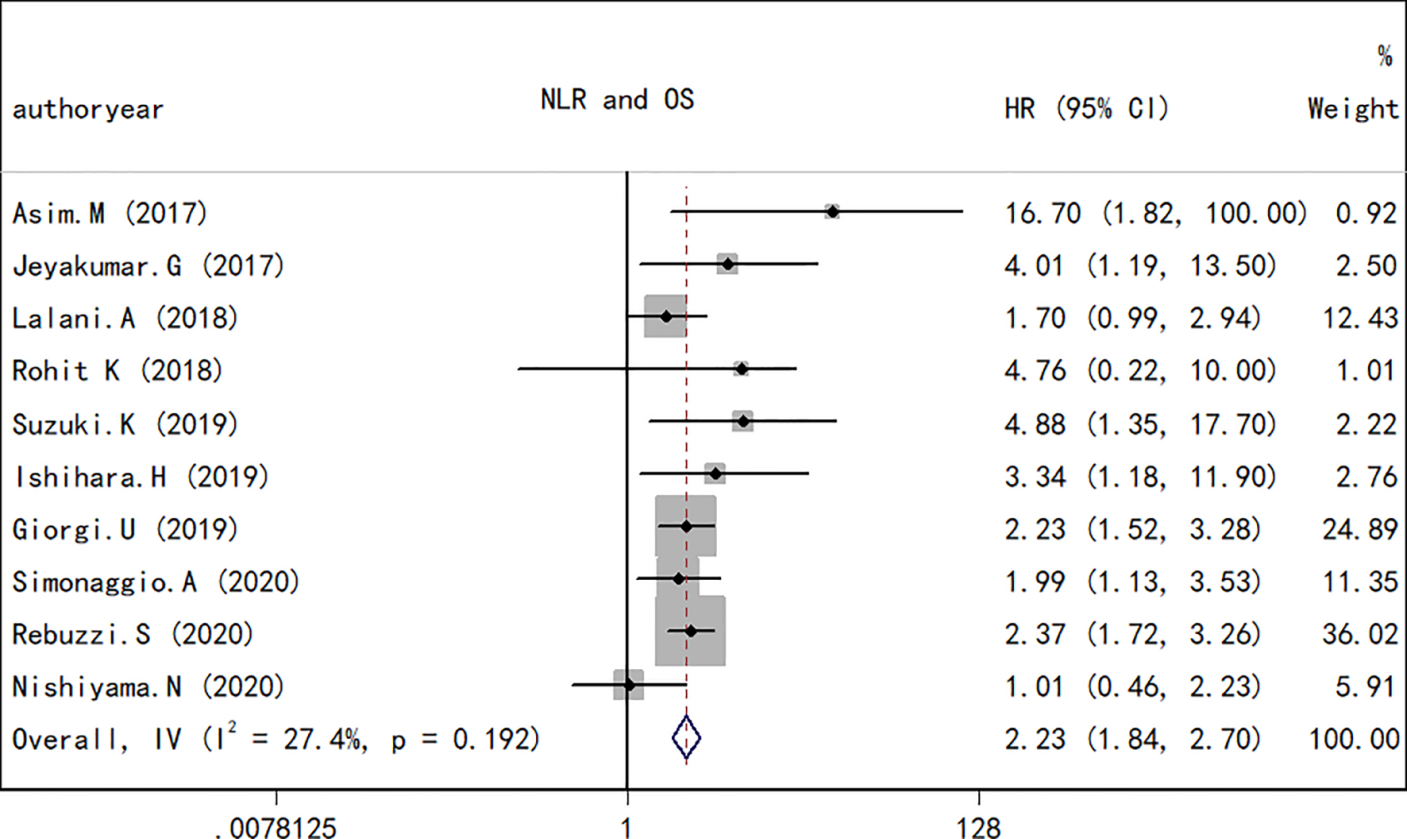
Forest plot of hazard ratios for the association between the neutrophil-to-lymphocyte ratio at baseline or pre-therapy and overall survival in renal cell carcinoma.

**Figure 3:**
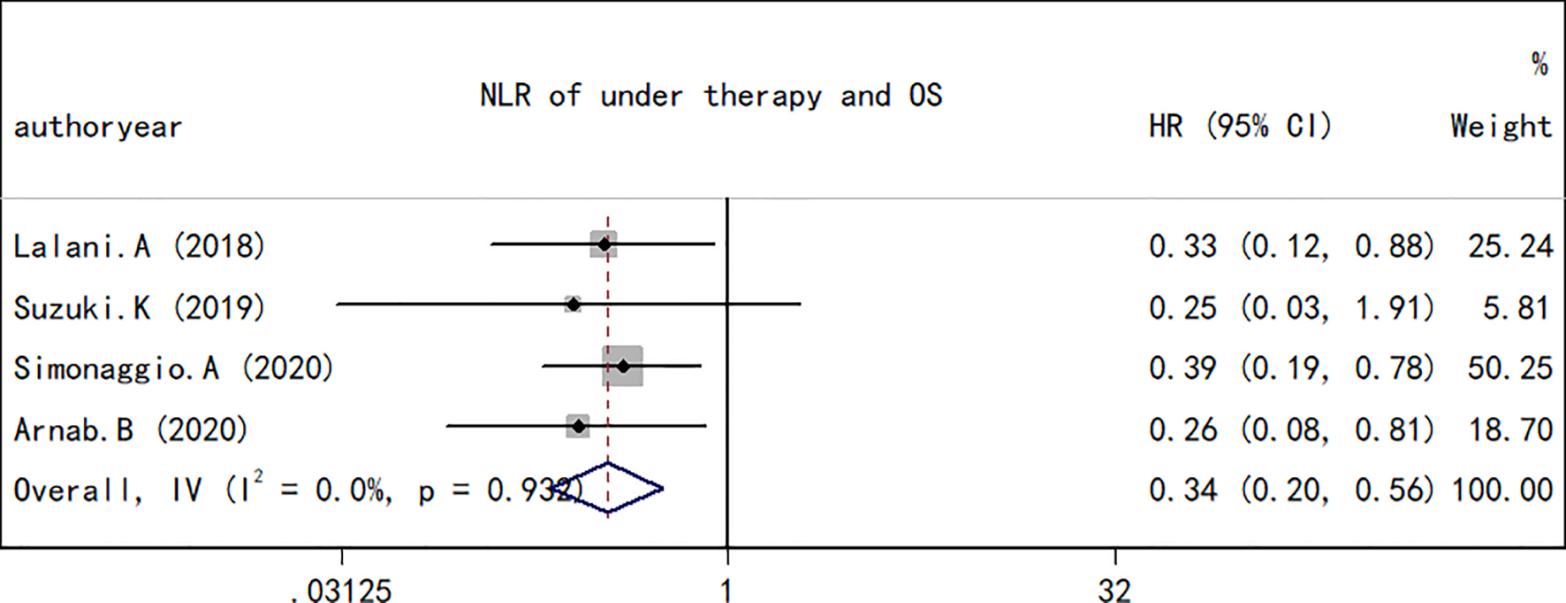
Forest plot of hazard ratios for the association between the neutrophil-to-lymphocyte ratio at baseline or pre-therapy and progression-free survival in renal cell carcinoma.

A total of 10 studies containing 1,148 patients reported the association between NLR at baseline or pre-therapy and OS in patients with RCC treated with ICIs. The random-effects model was used to estimate the combined HR and corresponding 95% CI values. The results revealed that the high NLR group at baseline or pre-therapy had a shorter OS than the low group (combined HR, 2.23; 95% CI, 1.84–2.70; *p* < 0.001; **Figure 2**), which suggested that a high NLR at baseline or pre-therapy was an important predictor of a poor prognosis. The *I* ^2^ (27.4%) and *Q* test (*p* = 0.192) results for OS indicated that there was no obvious heterogeneity among the studies. A total of four studies including 330 patients reported the effect of NLR during treatment on OS. The present study revealed that a decrease in NLR during treatment was a predictor of a longer OS (HR, 0.34; 95% CI, 0.20–0.56; *p* < 0.001; **Figure 3**). Correspondingly, the heterogeneity test revealed no statistically significant heterogeneity between the studies (*I* ^2^ = 0%, *Q* test *p* = 0.932).

### NLR and PFS in RCC

**Figure 4:**
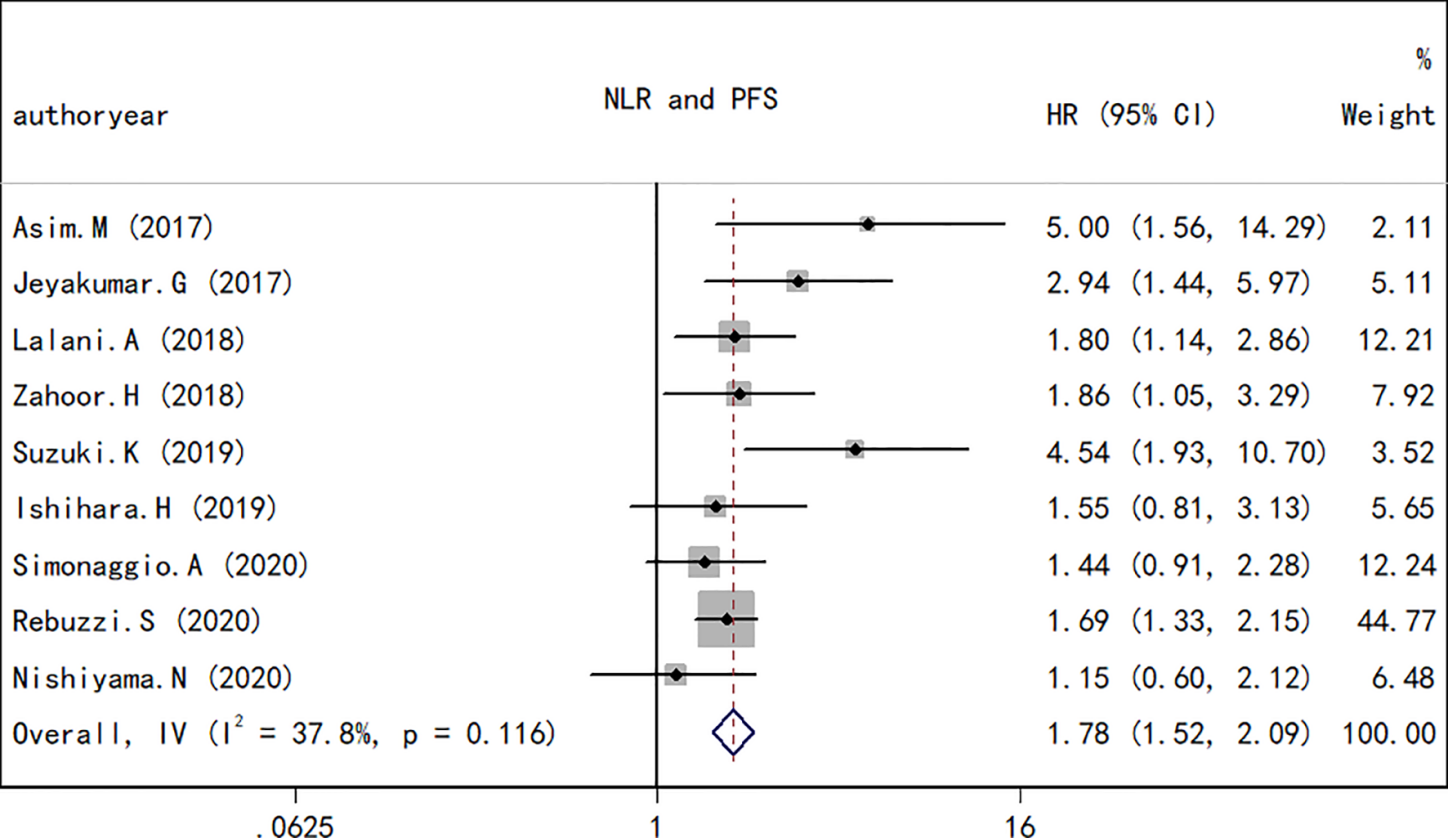
Forest plot of hazard ratios for the association between the neutrophil-to-lymphocyte ratio under therapy and overall survival in renal cell carcinoma.

**Figure 5:**
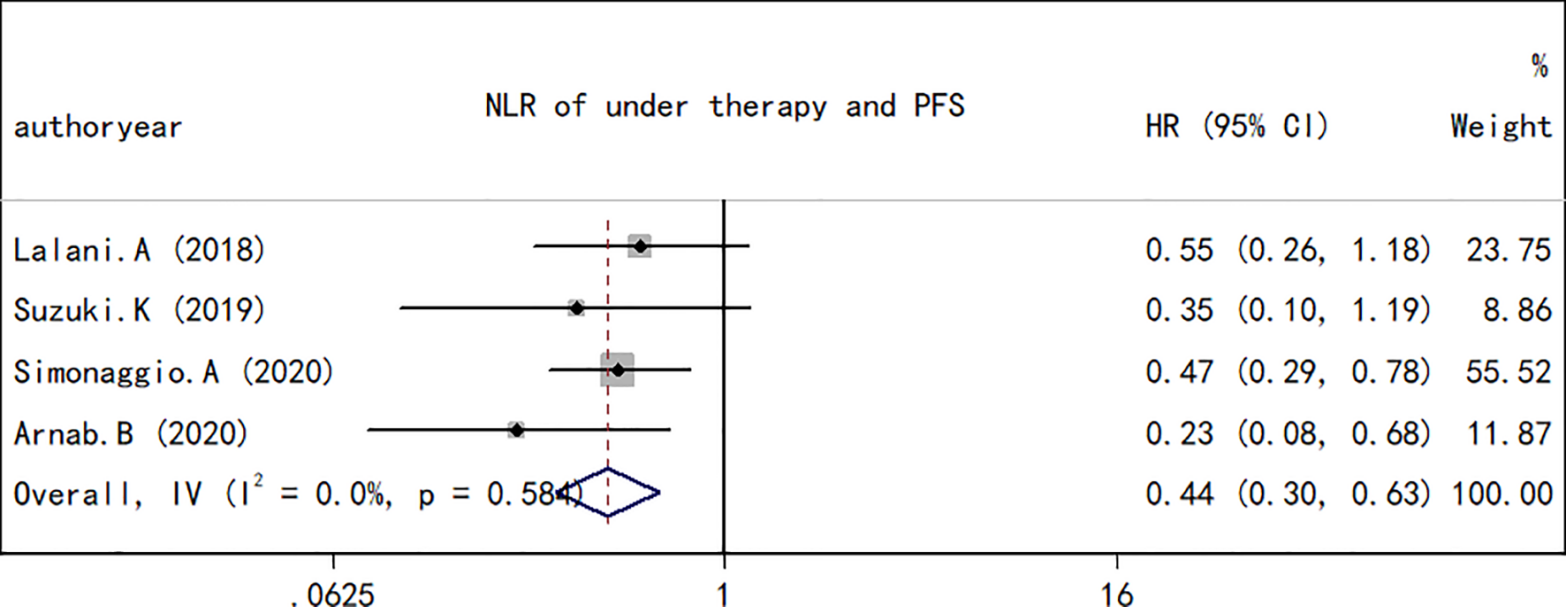
Forest plot of hazard ratios for the association between the neutrophil-to-lymphocyte ratio under therapy and progression-free survival in renal cell carcinoma.

As performed for the OS analysis, the association between the NLR at baseline or pre-therapy and PFS was estimated. A total of nine studies including a total of 1,173 subjects were used to investigate the clinical outcome, and a final combined HR of 1.78 (95% CI, 1.72–2.09; *p* < 0.001; **Figure 4**) indicated that a higher NLR was associated with a worse PFS in patients with RCC treated with ICIs. A low heterogeneity in the present analysis in terms of PFS (*I* ^2^ = 37.8%, *Q* test *p* = 0.116) was found. The association between changes in NLR during treatment and PFS was also explored. As observed in the OS analyses and in the PFS comparison, an increase in NLR during treatment resulted in a worse PFS (HR, 0.44; 95% CI, 0.30–0.63; *p* < 0.001; **Figure 5**) and no significant heterogeneity existed (*I* ^2^ = 0%, *Q* test *p* = 0.584).

### Other Risk Factors, and PFS and OS in RCC

The association between the efficacy of immunotherapy for mRCC and other possible risk factors including age, sex, race, histological type, ECOG status, prior nephrectomy, number of prior anti-VEGF therapies, IMDC risk group, and the number of metastatic sites was also explored. The results are presented in **Table 2**. Among all the risk factors, age, sex, race, and IMDC risk grouping may be prognostic factors for mRCC treated with immunotherapy. Males had a worse OS than females (HR, 1.48; 95% CI, 1.14–1.93); there was no significant difference in PFS between the two groups (HR, 1.10; 95% CI, 0.85–1.44). People over 70 years old had better OS compared to people younger than 70 (HR, 0.65; 95% CI, 0.48–0.89); however, there was no significant difference in PFS between the two groups (HR, 0.73; 95% CI, 0.51–1.06). Non-Caucasians treated with immunotherapy had a worse OS (HR, 8.67; 95% CI, 2.87–26.2) and PFS (HR, 2.65; 95% CI, 1.28–5.48) than Caucasians. Compared with the IMDC favorable risk group, the OS of the IMDC poor risk group was worse (HR, 2.59; 95% CI, 1.56–4.32); there was no statistically significant difference in PFS between the two groups (HR, 1.20; 95% CI, 0.74–1.94). Other risk factors including histologic type, ECOG, prior nephrectomy, number of prior anti-VEGF therapies, and number of metastatic sites did not affect the prognosis of patients treated with ICIs. However, the number of studies involving the prognostic value of these risk factors was too small. The results of the present study can be used as a preliminary screening of prognostic factors, and a more specific meta-analysis can be performed for further exploration in the future.

### Publication Bias

**Figure 6:**
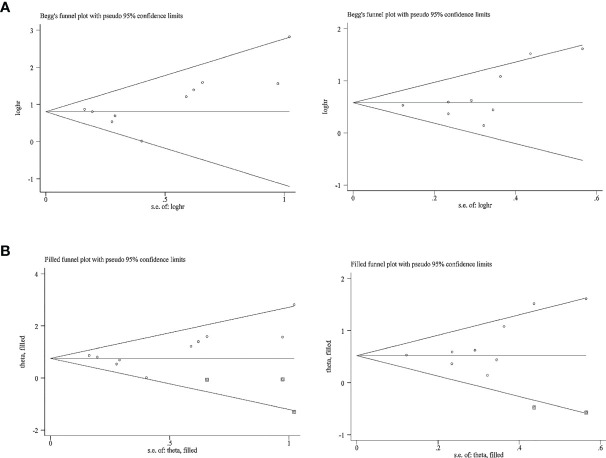
Funnel plot for the evaluation of publication bias considering the association between the neutrophil-to-lymphocyte ratio at baseline or pre-therapy and clinical outcomes in this analysis. **(A)** Funnel plot for 10 studies regarding overall survival and 9 studies considering progression-free survival before the “trim and fill” method. **(B)** Funnel plot for 10 studies considering overall survival and 9 studies considering progression-free survival after the “trim and fill” method.

The publication bias for OS and PFS was then assessed. In terms of the impact of NLR on the OS and PFS, Egger’s test revealed no obvious publication bias (*p* > 0.05); however, Begg’s test raised a high risk of publication bias (*p* = 0.05) in terms of the impact of NLR at baseline or pre-therapy on the OS and PFS (**Table 3**); funnel plots revealed a slight basic asymmetry by visual assessment (**Figure 6A**). To resolve this issue, a sensitivity analysis was implemented using the “trim and fill” method in STATA software, which removed or supplemented certain trials to examine the changes in the pooled effect size. If the conclusions were consistent, the publication bias was not obvious and the results were relatively stable. As far as OS was concerned, before the “trim and fill method”, the combined effect size Log (HR) and the corresponding 95% CIs were 0.80 (0.61–0.99). After three studies were supplemented, the pooled HR and 95% CIs were 2.13 (1.76–2.57). In terms of PFS, the effect size [Log (HR) and HR] and 95% CIs before and after “trim and fill” were 0.58 (0.42–0.74) and 1.78 (1.52–2.09), respectively. The above data showed that the conclusions were consistent, indicating that the result was stable. The imputed studies produced a symmetrical funnel plot (**Figure 6B**), which showed no publication bias; thus, the results were reliable in the current meta-analysis.

**Table 3:** Results of Egger’s and Begg’s tests for publication bias.

Outcome	Study number	Egger’s test (*p*)	Begg’s test (*p*)
OS	10	0.18	0.05
PFS	9	0.15	0.05
OS for under therapy group	4	0.15	0.31
PFS for under therapy group	4	0.35	0.73

## Discussion

As one of the inflammatory factor indicators, NLR can predict the efficacy of various therapeutic options, such as surgical resection and VEGR inhibitors in RCC (32, 33). The prognostic value of NLR in a variety of solid tumors undergoing immunotherapy has also been extensively explored (34, 35), and these findings suggest that a high NLR is a predictor of a poor survival in patients undergoing immunotherapy, which was consistent with the findings of the present study. Although the association between NLR and the prognosis of patients receiving immunotherapy has also been widely investigated in RCC, it remains a difficult task to determine the prognostic value of NLR in patients due to the small sample sizes of individual studies and the conflicting results of various studies. The present study provides strong evidence that NLR may be applied as a prognostic marker for patients with RCC receiving immunotherapy.

As the first meta-analysis (to the best of our knowledge) fully investigating the association between NLR and the prognosis of patients with RCC receiving immunotherapy, the present study summarized the available credible evidence from 12 studies encompassing 1,275 cases. The integrated HR confirmed that an elevated NLR at pre-therapy or at baseline was associated with a poor OS (HR, 2.23; 95% CI, 1.84–2.70; *p* < 0.001) and PFS (HR, 1.78; 95% CI, 1.72–2.09; *p* < 0.001). A significant association was also found between a decrease in NLR under therapy and an improved OS (HR, 0.34; 95% CI, 0.20–0.56; *p* < 0.001) and PFS (HR, 0.44; 95% CI, 0.30–0.63; *p* < 0.001). These results confirm that the NLR may be used as a prognostic indicator in patients with CC receiving immunotherapy.

Numerous studies have suggested that systemic inflammatory responses and the tumor microenvironment play a critical role in cancer progression and affect a patient’s response to treatment. At different stages of tumorigenesis, invasion, and metastasis, tumor cells and related inflammatory cells release a large amount of cytokines, chemokines, and other inflammatory factors to promote tumor initiation (10, 36). Thus, the systemic inflammatory response is significantly associated with the outcome of patients and related inflammatory indicators, such as the NLR, and this may be used as a biomarker for the prognosis of patients with cancer, and may effectively estimate the prognosis of these patients (37, 38). Neutrophils can promote cancer progression by directly acting on tumor cells or indirectly altering the tumor microenvironment (39). In the tumor microenvironment, neutrophils are separated into high-density neutrophils (HDNs) and low-density neutrophils (LDN) due to functional differences. LDNs suppress T cells through arginase expression and promote tumor angiogenesis by upregulating tumor vascular endothelial cytokines (VEGF), thus promoting tumor progression. Instead, HDNs function as antitumor agents either by acting directly on cancer cells or by provoking T-cell-mediated immune responses. In the context of inflammation, neutrophils primarily display an HDN phenotype in the early stages of inflammation, whereas the LDN phenotype is inclined to accumulate when the inflammation subsides (39, 40). As immune response cells, lymphocytes play a dominant role in the antitumor effect. Lymphocyte infiltration in tumor tissue is associated with a better therapeutic response and outcome, while the decrease in lymphocytes in the tumor microenvironment leads to a decrease in antitumor ability, which causes the emergence of immune tolerance and the escape of tumor cells (41). In addition, the reduction in peripheral blood lymphocytes can provide an appropriate tumor microenvironment for the proliferation and metastasis of tumor cells by impairing the antitumor response mediated by lymphocytes (42). Theoretically, neutrophilia represents the response to systematic inflammation, whereas lymphocytes reflect an impaired cell-mediated immunity. Therefore, a decreased NLR is associated with a better response to immunotherapy.

The predictive value of the NLR in the efficacy of immunotherapy for esophageal cancer, lung cancer, melanoma, and other solid tumors has been fully explored. As regards esophageal cancer, the PFS in patients with a high NLR at 6 weeks post-treatment was shown to be lower than that of patients with a low NLR (HR, 2.097; 95% CI, 0.996–4.417; *p* = 0.027) (16). An elevated NLR at pre-treatment has been shown to be associated with a shorter OS and lower response rates in patients with metastatic NSCLC treated with Nivolumab independently of other prognostic factors (34). In another study, a similar association was observed between the NLR and the efficacy of Ipilimumab in the treatment of melanoma (35). All the aforementioned independent studies confirmed the prognostic value of NLR in immunotherapy; however, these individual studies were retrospective studies and the sample sizes were small.

Another two high-quality meta-analyses also explored the association between NLR and the survival of patients with solid tumors treated with immunotherapy. A previous meta-analysis included 27 studies incorporating 4,647 patients with advanced cancers consisting of non-small cell lung cancer (NSCLC), RCC, and hepatocellular carcinoma, among other types. The pooled analyses indicated that a high blood NLR at pre-therapy was associated with a significant shorter OS (HR, 1.98; 95% CI, 1.66–2.36; *p* < 0.001) and PFS (HR, 1.78; 95% CI, 1.48–2.15; *p* < 0.001). In that study, immunotherapy was defined as a form of treatment that acted on the immune microenvironment, including CTLA-4, PD-1, PD-L1, VEGF, and VEGFR, among other targets, and the involved patients with RCC were all treated with Sunitinib and Sorafenib (43). However, in the present study, the patients with RCC were treated with ICIs, including CTLA-4, PD-L1, and PD-1. In another meta-analysis, seven studies were included, containing three trials on melanoma, three studies on NSCLC, and only one study on RCC. The pooled results revealed that a high NLR contributed to a worse OS (HR, 1.92; 95% CI, 1.29–2.87; *p* < 0.001) and PFS (HR, 1.66; 95% CI, 1.38–2.01; *p* < 0.001) (44). Although both studies assessed the prognostic value of NLR in patients with malignant tumor receiving immunotherapy, the predictive significance of NLR in patients with malignant tumors receiving immunotherapy remains unknown due to differences in the definition of immunotherapy and fewer studies involving RCC. In the present study, the prognostic value of NLR in patients with RCC receiving ICIs was comprehensively identified. The pooled results showed that a high NLR was significantly associated with a poor OS and PFS, indicating that blood NLR was a significative predictive biomarker in patients with RCC receiving immunotherapy.

However, the present study has several limitations. Firstly, Egger’s test indicated that a slight publication bias was present; although a “trim and fill” analysis was conducted, the combined results should be treated with caution. Secondly, the current meta-analysis is a literature-based analysis rather than individual patient data-based analysis, which renders the results less reliable. Thirdly, studies that could not provide sufficient information to calculate the HR were excluded, which would cause the combined effect size to differ from true values to a certain extent. Considering these factors, further more robust analyses are required to verify or update these results in the future.

In conclusion, in view of the current meta-analysis, the results revealed that a high blood NLR was associated with a poor OS and PFS across studies of patients with RCC treated with ICIs. Therefore, the NLR may be used as a prognostic indicator for patients with RCC accepting ICIs based on available trials, which may help to direct clinical decision-making. Nevertheless, future prospective randomized controlled trials are required to confirm and better understand this biomarker and its role in the employment of ICIs in RCC.

## Data Availability Statement

The original contributions presented in the study are included in the article/supplementary material. Further inquiries can be directed to the corresponding author.

## Author Contributions

XC and RJ designed and performed the study, and wrote the manuscript. FM extracted the data, and assisted in data collection and analysis. XC and FM critically revised the manuscript, and ensured correct data analysis. RJ and XC confirmed the authenticity of all the raw data. All authors contributed to the article and approved the submitted version.

## Funding

The present study was funded by the Nature Science Foundation of Tianjin City (grant no. 18JCZDJC98800).

## Conflict of Interest

The authors declare that the research was conducted in the absence of any commercial or financial relationships that could be construed as a potential conflict of interest.

## Publisher’s Note

All claims expressed in this article are solely those of the authors and do not necessarily represent those of their affiliated organizations, or those of the publisher, the editors and the reviewers. Any product that may be evaluated in this article, or claim that may be made by its manufacturer, is not guaranteed or endorsed by the publisher.
